# The Education of the Defective in Canada

**Published:** 1915-05-29

**Authors:** 


### THE EDUCATION OF THE DEFECTIVE IN CANADA.

### The Benefits of Auxiliary Classes.

Despite the most strenuous efforts of the eugenists it is certain that there will be possibly for centuries to come many members born into all communities whose lives as normal, self-supporting individuals will be blighted by defect. There are still some people who would adopt the heroic measures of earlier times with these unfortunates, but such drastic treatment is, however, fortunately a matter for academic discussion rather than for practice. In the meantime the problem is rather how such afflicted beings may be enabled to live happily, if not usefully; and, if possible, how both these much-to-be-desired ends may be attained. The generality even of scientific people when faced with such a problem are apt to fold their hands helplessly and say that it is Fate—and leave the matter there. To such despairing folk the lessons embodied in the lives of, for example, Laura Bridgman and Helen Keller may well be commended; and if these do not serve to render them more optimistic they should study the plain, straightforward statements in Dr. Helen MacMurchy's report on the "Organisation and Management of Auxiliary Classes."*

Dr. MacMurchy has not only dealt with the conduct of these classes as they have come within her purview as Inspector of Auxiliary Classes for Ontario; she has gone further afield and has brought together information of what has been done and what is being done for defectives in many other parts. In this connection it is gratifying to note that, according to Dr. MacMurchy, "more has been done in England for physically defective children than in any other country." That more remains to be done goes without saying; and the discussion which preceded the passing of the Mental Deficiency Act of 1913 made it abundantly clear that much strenuous labour is required. Unfortunately the problem of dealing adequately with the feeble-minded has had to be shelved until the *sans-culottes* of Europe are put under restraint! It is a matter for congratulation that even the present distressful conditions cannot interfere radically with the carrying on of this beneficent and useful work; and it is to be hoped that even in Germany this may be equally the case. Much of the early work in the provision of auxiliary classes was done in Germany; as long ago as the year 1859 Professor Haupte, of Halle, began a special class for defective children. Prior to this, however, private schools for imbeciles had been instituted in England at Bath and Highgate.

Mental defectiveness, as everyone is aware, is a matter of degree. The individual may be so deficient as to be classed in the category of idiots. On the other hand the defect may be so slight in degree as to be unascertainable except by scrupulous and expert examination. For instance, where the defect is of the nature described as "moral," the intelligence may be of a higher standard than the average exhibited by the patient's compeers, and yet the patient is found to be deficient in certain respects. He is a source of evil to others who associate with him, he is a thorn in the flesh of those who try to educate him in ordinary classes, and he may be—or may become—a pest to society. It is important to recognise at an early date, if possible, whether this condition is really due to some inherent organic defect or whether the perverse characteristics are the result of faulty environment. If it is due to defective organisation it is obvious that the individual can never be educated to the normal standard. Again, the defect may not be in the central nervous system, though it may appear to be so. The brain can only be reached through the senses; consequently if the senses are defective the person is handicapped in the acquisition of knowledge. Therefore the child with defective vision, for example, must have allowance made for him. Now, if such children are members of ordinary school classes they are either left behind by their more easily educable neighbours or, if perchance the teacher endeavours to keep them up to the average of knowledge in the class, they serve as a drag upon the whole class.

Special classes not only obviate these undesirable contingencies: they make it possible that these examples of unfinished mankind shall be fashioned into comparatively useful members of society. Some there are, of course, whom no amount of special training can bring to this desirable standard; and it is important that this should be definitely ascertained in order that further educative efforts may not be wasted on a hopeless task. A perusal of such a report as that which Dr. MacMurchy has written will make it clear, however, that even apparently hopeless cases may be modified in an almost amazing way. A visit to such an establishment as Darenth, or the reading of the small but concise and compact volume by Mr. Bickmore, the craftsmaster at that institution, in which he describes the industries which are carried on by the feeble-minded and imbecile population there, will add to the strength of anyone's belief in the efficacy of the measures which have been adopted.

One desideratum in dealing with the feeble-minded portion of the community in whom actual defectiveness has been definitely ascertained is that they should be segregated during the whole of their lives. Misplaced sentiment and false economy too often militate against the effective carrying out of what seems at first a somewhat Draconian edict. The community is only fulfilling an obvious duty when it affords special protection and training to the mentally defective. On the other hand the community should, in the words of Dr. MacMurchy, "be protected from the expense, immorality, crime, and national degeneration caused by them and by the posterity they bring into the world unless they are under permanent and proper care."

* Published by the Department of Education, Ontario.

